# Evolution over 11 years of the characteristics of walk-in patients at the emergency department of a university hospital in Brussels

**DOI:** 10.25122/jml-2018-0053

**Published:** 2019

**Authors:** Merita Hysenbegasi, Ives Hubloue, Rita Vanobberghen, Jan Kartounian, Dirk Devroey

**Affiliations:** 1. Department of Family Medicine and Chronic Care, Vrije Universiteit Brussel, Belgium; 2. Department of Emergency Medicine UZ Brussel and Research Group on Emergency and Disaster Medicine, Vrije Universiteit Brussel, Belgium

**Keywords:** Emergencies, emergency department, family practice

## Abstract

Walk-in patients who do not require urgent treatment at an emergency department (ED) are a known and long-standing problem.

This study aims to investigate the characteristics of walk-in patients visiting the ED over time.

During four days in June 2012, all walk-in patients attending the ED of the University Hospital Brussels between 8 AM and 11 PM were recorded. A similar registration took place in the same ED in June 2001. Patients completed a questionnaire about their characteristics and the reason for the encounter. Data of both study periods were compared.

The mean age of the patients attending the ED was significantly lower in 2001 (40.9 years) than in 2012 (43.9 years) (p=0,02). In 2001, 81% of the participants had Belgian nationality, but in 2012 this proportion increased to 90% (p=0.008). In 2001 as well as in 2012, 21% of the participants had a referral from their family physician (FP) (p=0.9). The proportion of patients that were aware that FP could also handle some emergencies increased from 17% in 2001 to 29% in 2012 (p=0.003). More patients had complaints that begun less than 24h before they attended the ED (48% in 2001 and 58% in 2012) (p=0.03).

The walk-in patients at the ED are getting slightly older and are attending the ED faster after the onset of the complaints. More patients judge their complaints as urgent. However, more patients are getting aware that FP also could handle some emergencies.

## Introduction

In Belgium, patients can attend emergency departments (ED) and specialists without a referral from their family physician (FP). Nevertheless, because of their long-term and in-depth relationship with their patients, FPs are well positioned to take care of some emergency-demanding conditions for which a primary care approach is appropriate.

Several studies show that many patients attend EDs without prior contact with their FP or with a disease which can be treated in primary care [1–4]. Improper use of ED threatens the timely treatment of severe and life-threatening conditions of other patients. There are several reasons for this: threshold too low for ED created by media and television series, the difficult access to the FP, the overvalued opinion of a specialist, the lower threshold for more examinations, a second opinion, a lack of a recognizable localization of the FP on duty, patients without FP, poor understanding of the notion of “emergency”, a lack of knowledge about the health care system (recent migrants, disadvantaged), financial reasons and so forth [1,5].

### Continuity of care

Emergency care is more than a solution to guarantee the continuity of care. In Belgium, continuity of care is a legal guarantee available to all persons in need of medical care, regardless of their FP and their situation. The FPs are organized in local associations of FPs. The main task of these local associations is to organize the local non-plannable care which includes conditions for which an urgent primary care approach is possible. The local FPs are staffing this local, non-plannable care. This care was in the previous decennia organized in the office of the FP “on duty”. Since 2003, more and more local associations install out-of-hours primary care wards where patients can attend at a fixed place for non-plannable medical care. The local associations make arrangements with hospitals and specialists to optimize this care.

### Preference for hospital care

A majority of patients prefer emergency hospital care above emergency primary care. In several countries, there are triage systems to overcome this problem. The importance of the triage system was demonstrated in a study in London, showing that 41% of patients attending the ED needed a primary health care approach [2]. A study in Finland shows that a triage system may achieve a shift in emergency care from the public sector to the private sector, but no changes in the number of patients that presented themselves to the ED was achieved [3]. Most patients attending the ED without referral consider the ED to be the most suitable place to manage their problem because of the diagnostic facilities and the belief that hospitals are better qualified to treat their problem [5–8]. A second opinion from a specialist can also be a reason to choose an ED [6,9].

### Problems with primary care

Studies in Switzerland and The Netherlands show that the majority of the walk-in patients did not have an FP [6,10]. This is mainly a problem among younger patients [6]. Limitations in the availability and accessibility of comprehensive primary health care encourage patients to attend ED as an alternative to primary health care [11]. Better access for patients with non-plannable conditions in primary care can reduce in the ED the number of patients with conditions that can be treated in primary care [12]. Dissatisfaction with loco-regional out-of-hours primary care wards was also one of the reasons to prefer ED [5].

### Age

In the US, older adults who live alone have a preference for the ED [13]. In Australia, older patients with non-urgent diseases also prefer the ED rather than their FP [14]. For the elderly, the proportion of visits to ED can be reduced by increasing the availability of primary health care [15]. In Switzerland, the majority of walk-in ED attendees were younger patients [10].

### Cultural identity

Patients of foreign nationality present themselves considerably more in the ED. They have less knowledge about the accessibility and organization of “out of hours” services [10,16]. They often prefer an ED over an FP because of their limited access to insurance. However, the increased use of ED by immigrants can also be related to their younger age and occupations with a higher risk of accidents [17].

### Financial reasons

In most cases, when visiting the FP, the patient must immediately pay the entire consultation fee. At the ED, the patients pay only a small amount of the consultation fee while their insurance will pay the remaining amount. For people who have financial difficulties, this seemingly lower fee plays an important role [18].

In Belgium, a low socio-economic level correlates with more hospitalizations and more visits to the FP and nurses. People with higher education are more often registered with a specialist, a physiotherapist and a dentist [19].

### Perception of the urgency of care

A poor understanding of the meaning of “emergency” is one of the main reasons for consulting the ED. According to the patients, “emergency” means serious and the need for urgent examinations [20]. Anxiety about their disease (poor perception), the need for a second opinion and the lack of medical knowledge are the main reasons why patients attend the ED [21]. Patients do not need to be blamed for the difficult choice between urgent and non-urgent care or the difficult choice between primary care and emergency care. The degree of urgency is recognized as extraordinarily difficult to objectivate, especially for the patients themselves. Since 2013, an “out-of-hours phone number” has been launched in Belgium to help patients to find the most appropriate care. Facilitating patients in their search for medical care is essential but also a better understanding of their health-seeking behavior is needed.

### The aim of the study

Since 2003, emergency care in Belgium is better-organized thanks to a better organization of the local associations of FPs and the installation of loco-regional out-of-hours primary care wards. These actions may have an influence on the consultation behavior of patients attending the ED.

This study aims to investigate the characteristics of walk-in patients visiting the ED and whether these characteristics changed over time. Special attention was paid to the reason for the encounter and the referral by FP.

## Materials and Methods

### Patient selection

We recorded all adult walk-in patients who attended the ED of the University Hospital Brussels during a 4-day period (Friday to Monday) in June 2012. Only patients attending between 8 AM and 11 PM were included.

Patients who arrived in an ambulance were excluded because they already passed through a triage filter and their medical condition was probably urgent enough to justify a visit to the ED. These are the same inclusion criteria which were used for a similar registration in the same hospital in 2001 [22].

### Questionnaire

Every patient who met the inclusion criteria of the study was interviewed. The interviewer completed a questionnaire (see appendix) including demographic information, nationality, profession, the reason for encounter, the reason to attend the ED, information about the FP and subscription with an FP, knowledge about loco-regional out-of-hours primary care wards. The diagnosis was added to the questionnaire at the end of the visit to the ED. All interviews were done by one interviewer (MH). She was at the time of the study a trainee in family medicine. She followed training on conducting interviews.

### Medical ethics

The Ethics Committee of the University Hospital Brussels approved the study protocol. Each patient received information about the study and signed informed consent. Confidentiality and anonymity were guaranteed.

### Statistical analysis

IBM SPSS Statistics 20 was used for analysis and statistical processing. For continuous variables, the Student t-test was used to compare the means between groups. For discrete variables, the Chi-square test was used to detect significant differences between groups. The statistical analyses were initially performed by IH and DD. In the second stage, the other authors were involved in the statistical analyses.

## Results

### Patients characteristics

In 2001 and 2012, 208 and 232 walk-in patients were enrolled in the study, respectively. In 2012, 18 patients refused to participate while for 2001 there were no available figures about refusal. For the registration in 2012, the distribution over the shifts was available for the 4 registration days (Table 1). For the 2001 registration, these figures were not available. The busiest shifts were on Sunday afternoon and Monday morning. The number of patients per hour was the highest in the morning.

**Table 1:** Number of registrations according to the day of the week and period of the day

	Friday	Saturday	Sunday	Monday	Total	Patients/h
**8–12 h**	19	14	18	29	80	20
**12–18 h**	21	22	28	21	92	15
**18–23 h**	19	13	11	17	60	12
**Total**	59	49	57	67	232	15

The mean age of the patients attending the ED in 2001 (40.9 years) was significantly lower than the mean age of patients attending in 2012 (43.9 years) (p=0,02). In 2001, 51% of the participants were female. In 2012, this proportion increased to 58%, but the increase was not significant (p=0.18).

In 2001, 81% of the participants had Belgian nationality. In 2012, this proportion increased significantly to 90% (p=0.008). Other frequently attending nationalities in 2012 were Moroccan (n=5), Polish (n=3), Romanian (n=3), Spanish (n=3), Greek (n=2), Italian (n=2) and Dutch (n=2).

In 2012, some additional characteristics of patients were recorded. The language during the interview was for most of the patients French (70%), followed by Dutch (28%) and English (1%). The remaining 1% spoke another language. For them, a family member or a member of the staff acted as translator.

Most of the participants were married (48%), 35% lived alone, 9% were divorced, 6% were widow(er)s and 2% lived together without being married. Most of the participants were employees (26%), followed by laborers (21%), retired (18%), unemployed (13%), self-employed (10%), students (9%) and disabled (3%).

### Family physicians

In 2001, 84% of the participants had an FP. In 2012, this proportion was 85%, but the increase was not significant (p=0.624). In 2001, 30% of the participants had contact with their FP before they attended the ED. In 2012, this proportion was 28%, but the decrease was not significant (p=0.18). There was no significant difference in the proportion of patients that were referred by their FP to the ED. In 2001, as well as in 2012, 21% of the participants had a referral from their FP (p=0.9).

The proportion of patients aware that FPs can also handle some emergencies such as some wounds and musculoskeletal injuries increased from 17% in 2001 to 29% in 2012 (p=0.003).

In 2012, some additional characteristics of FPs were recorded. 72% of the patients were registered with an FP. Only 42% of the attending patients know that they can also attend an FP for emergencies in a primary care emergency ward. Because these data were not recorded in 2001, the comparison between both registrations is not possible.

### Emergency department

The proportion of patients attending with complaints that begun less than 24h before they attended the ED increased from 48% in 2001 to 58% in 2012 (p=0.03) (Table 2). For the patients with complaints that began between ‘24h and 1 week’ earlier and for complaints that started more than one week before the visit to the ED, no significant differences were observed. Overall, patients did not go to the ED more quickly (p(2)=0.09).

**Table 2:** Duration of the complaints before attending the ED

	2001 (n=208)	2012 (n=232)	P-value
**Less than 24h**	48%	58%	0.03
**24h to 1 week**	34%	29%	0.24
**More than 1 week**	18%	13%	0.16

The proportion of patients that judged their complaints as not severe remained stable over both registration periods (Table 3). In 2001, one-quarter of the patients could not judge the severity of the complaints. This proportion has decreased resulting in an increase of the patients judging their complaints as urgent. Overall, a change in the severity of the complaints as observed by the patients was recorded (p=0.001).

**Table 3:** Severity of the complaints of patients attending the ED

	2001 (n=208)	2012 (n=232)	P-value
**Severe**	44%	59%	0.001
**Not severe**	30%	35%	0.25
**No answer**	26%	6%	<0.001

The proportion of patients that found access to the ED easier than access to the primary care emergency facilities was 54% in 2001 and 58% in 2012. However, the difference was not significant (p=0.4).

In 2012, some additional characteristics of FPs were recorded. 52% of the participants declared that they were not informed by their FP about the criteria to attend the ED.

In total, 21% attended the ED because their FP referred them to the ED. The ED was preferred above the primary care emergency ward by 79% because participants declared that the ED guarantees better examinations (16%), more experienced physicians (11%), better service (9%), shorter waiting time (7%), the complaint was too severe (7%), better treatment (4%), better accessibility (4%). The remaining reasons (21%) included better service, the possibility for hospitalization, less expensive, better examinations and others.

Diagnoses were coded according to the International Classification of Primary Care (ICPC). Most of the patents attended the ED for loco-motoric problems (37%). Skin problems are also very popular (12%), followed by general problems (9%), digestive problems (8%), respiratory problems (6%), urinary tract disorders (5%), digestive problems (3%), neurological problems (3%), circulatory problems (3%), problems of the blood and blood-forming organs (3%) and eye problems (2%). The other problems accounted for 1%. For 8% of the participants, the diagnosis could not be made at the ED.

## Discussion

The average walk-in visitor of the ED is a middle-aged (43 years old) Belgian married patient. Most of the patients have a job. Although 85% of the walk-in patients of the ED have an FP, most of them attend the ED without first consulting their FP. Having an FP does not seem to be a guarantee not to walk-in to an ED. Muller et al. found that 82% of the walk-in patients have an FP [23]. Other studies confirm that most of the walk-in patients have an FP [8,10,12]. Half of the walk-in patients do not know that out-of-hours primary care wards exist. This will probably have an influence on their care-seeking behavior [24].

Most of the walk-in patients choose the ED because of estimated better examinations, more experienced physicians and better service. Several studies confirm that patients expect better qualifications and better diagnostics facilities in ED as compared to out-of-hours primary care wards [6–9, 23].

Often, the disease is estimated severe by the patient and is the reason to attend the ED immediately. Poor perception of the severity of the disease is often mentioned as a reason for the inappropriate use of the ED [1,23,25–27].

Despite the fact that we knew the reason for encounter and the tentative diagnosis, it remains challenging and delicate to draw conclusions on the inappropriate use of the ED. The appropriateness should also take into account some socio-cultural and personal aspects such as the (out-of-hours) accessibility of primary care and the patients capacity to estimate the degree of emergency.

Most of the patients are not well informed about the skills of their FP. However, it is not certain that all FPs have the necessary skills to adequately manage musculoskeletal injuries and complex wounds.

Wounds need proper evaluation with an assessment for neurovascular damage, tendon and vascular involvement and may require specialist intervention if found to have these issues. Conversely, many superficial wounds can be closed without suturing. Potential fractures need radiological evaluation which is not usually done by FPs and would require a visit to an outpatient radiology department. Then, many can be managed without plaster casts and conversely some need a closed reduction in the ED and/or orthopedic review for potential operative management.

Only half of the patients are informed by their FP about the indications to visit an ED. Much evidence proves that better patient information can reduce the inappropriate use of the ED [25,28,29]. Information about the indications to visit an ED could in the future be distributed by means of leaflets in the waiting rooms of EDs and FPs. Repeated media campaigns could also contribute to the more appropriate use of EDs.

### Evolution over the past 11 years

There are a few remarkable differences between the 2001 and 2012 figures.

In 2012, the mean age of the patients was lower than in 2001, and the proportion of Belgian patients increased. However, 40 vs. 43 years old has no clinical significance even though it has statistical significance. A remarkable change is that in 2012 more patients are aware that FP can also handle some emergencies such as the management of some wounds and musculoskeletal injuries.

Another significant change is the fact that more patients attend the ED with complaints that begun less than 24 hours before their visit to the ED. On the one hand, this could be a good evolution for real emergencies such as myocardial infarctions or stroke, where it is desirable to attend the ED as soon as possible. However, the time of onset is not necessarily a reflection of emergency care need. For example, pneumonia can take a few days or more before requiring emergency care. On the other hand, we should notice that, in our study, walk-in patients mainly came to the ED within 24 hours after the onset of the symptoms. Most of these patients attended the ED without a referral from their FP. In that perspective, the faster visits to the ED could be a sign that patients prefer the ED above their FP for self-perceived emergencies for which they expect fast medical advice. This is confirmed by the fact that the proportion of the patients judging their complaints as being urgent increased in 2012.

We cannot expect that all walk-in patients should be referred by their GP. Some walk-in patients clearly require urgent treatment (e.g., wrist fracture requiring reduction, mental health crisis). However, for some patients, it is evident that they have a low acuity degree for emergency care. Because of patient empowerment and increased health literacy, patients should be better able to assess for themselves which care provider can help them best (e.g., the emergency department, FP and others).

Most patients find access to the ED easier than to the primary care out-of-hours facilities and this has not changed over the past 12 years. This can be explained by the fact that in the Brussels Capital Region, where the study took place, the out-of-hours phone number was not yet in use and because the out-of-hours primary care wards were not yet fully rolled out. Both initiatives should make access to primary care out-of-hours facilities easier.

### Strength and weaknesses

This small study covers only a 4-day period of the walk-in patients attending the ED of one hospital. The strength of this method is that we can compare it with a similar registration of the same length and in the same hospital.

Despite the diagnose and the grade of emergency that were reported, we were not able to link both in an indisputable way. The link between both might elucidate much of the behavior of the studied patients.

Unfortunately, the final diagnosis for the patients presenting at the ED was not always made at the ED. It could have been interesting to estimate the acuity degree for emergency care of the walk-in patients. Thereupon, the diagnosis made at the ED was sometimes adjusted during the subsequent hospitalization or ambulant consultations. These adjusted diagnoses were not recorded in the study. Without this information, it was difficult to evaluate whether the patient could have been safely managed elsewhere.

The reason why patients had difficulties in accessing (out-of-hours) primary care services were not really explored. A more in-depth qualitative study on why they chose the ED would be of interest.

Two out of four study days were weekend days. During weekend days, FPs are not available in their practice. That may increase presentations to the ED for primary care related issues. However, patients had access to the FP via the out-of-hours primary care wards.

It might be expected that we would also compare the absolute number of patients attending the ED. However, the changing demographics in Brussels (the population is growing very fast) and several other biases do not allow to compare these figures.

### Importance of the study

To be able to lower the number of unjustified self-referrals, different strategies should be combined. Various triage systems such as telephone triage, triage by nurses at the ED and triage by out-of-hours primary care wards (or the FP on duty) should work together more efficiently.

A well-organized gatekeeper system at the entrance of the ED can distinguish patients who belong at the ED and those who should attend their FP. This might cause a problem for patients without an FP, but it should stimulate them to find an FP and to visit the FP before attending the ED from then on.

Better accessibility and organization of the out-of-hours primary care wards and the FP on duty can also contribute to a better selection of patients. It has been suggested that higher personal financial contribution of the patients might also retain them to visit the ED [18]. However, we should note that such an action might hinder the accessibility of the ED for the poor and deprived.

It seems of the utmost importance that the patients are better informed about the indications to visit the ED and about the skills of their FP. Such information could be provided in the media or by leaflets in the waiting rooms of FPs or EDs.

## Conclusions

Walk-in patients attending the ED without a referral are getting slightly older and are attending the ED faster after the onset of the complaints. More and more patients judge their complaints as urgent. The most important positive finding in this study is the fact that more patients are getting aware that FPs can also handle some emergencies. A campaign should be launched to make the patients aware of the availability of out-of-hours primary care wards and the possibilities of FPs to treat emergencies.

Patients still encounter difficulties to judge whether their condition is severe or not. For a fractured wrist the diagnosis is easy but regarding chest pain, for example, remains difficult for the patient to assess the degree of urgency.

## Acknowledgment

The authors would like to thank all participants, as well as the staff members of the emergency department who contributed to the study. Special thanks to Els Huybregts for the excellent coaching and David Proot for the language editing.

## Conflict of Interest

The authors confirm that there are no conflicts of interest.

## Appendix

### Questionnaire

**Table:**

Date:	…………………
Time of arrival :	............
Age:	………… years
Sex:	□ men □ women
Zip code:	…………
Nationality:	…………
Marital status:	…………
Profession:	□ student
	□ unemployed
	□ labourer
	□ employee
	□ self-employed
	□ retired
	□ others …………

Main complaints for which you come to the emergency department:

…………………………………………………………………………………………………………

…………………………………………………………………………………………………………

For how long have you had these complaints?

 □ Less than 24 hours

 □ 24 hours to 1 week

 □ More than 1 week

Do you have an FP?

 □ Yes  □ No

Does your FP have your personal medical record?

 □ Yes  □ No

Have you contacted an FP before your visit to the ED?

 □ Yes  □ No

Have you been referred by an FP?

 □ Yes  □ No

Was it easier to come to the ED instead of the FP?

 □ Yes  □ No

Do you think that an FP is able to stitch a wound?

 □ Yes  □ No

Has your FP ever informed you that he is also doing stitches?

 □ Yes  □ No

Has your FP ever informed you how you can reach him outside working hours?

 □ Yes  □ No

Are you informed about the primary care out-of-hours wards?

 □ Yes  □ No

Has your FP ever informed you when you should immediately go to an ED?

 □ Yes  □ No

Did you find your complaints too serious to be treated by an FP?

 □ Yes  □ No

Would you attend an FP if you knew that he could treat your complaints as well as the ED?

 □ Yes  □ No

Why did you come to the ED?

 □ more experience

 □ better service

 □ less expensive

 □ shorter waiting times

 □ more additional investigations

 □ better treatment

 □ possibility of hospitalization

 □ accessibility

 □ anonymity

 □ second opinion

 □ too serious complaints

 □ other:……………………………………

Comments: ………………………………………………………………………………

Final diagnosis: ………………………………………………………………………………
